# Endogenous Metabolites Released by Sanitized Sprouting Alfalfa Seed Inhibit the Growth of Salmonella enterica

**DOI:** 10.1128/mSystems.00898-20

**Published:** 2021-02-09

**Authors:** Ga-Hee Ban, Yue Dai, Tao Huan, Alfred Ke, Pascal Delaquis, Siyun Wang

**Affiliations:** a Food, Nutrition and Health, Faculty of Land and Food Systems, The University of British Columbia, Vancouver, British Columbia, Canada; b Department of Chemistry, Faculty of Science, The University of British Columbia, Vancouver, British Columbia, Canada; c Agriculture and Agri-Food Canada, Summerland Research and Development Centre, Summerland, British Columbia, Canada; Teagasc Food Research Centre

**Keywords:** metabolomics, alfalfa, *Salmonella enterica*, sanitizer

## Abstract

Sprouts are the leading cause of foodborne disease outbreaks globally, mainly because the specialized conditions required to germinate seed sprouts for human consumption contribute to an environment that allows pathogenic bacteria to flourish. To reduce risk of illness, current food safety guidelines in the United States and Canada recommend hypochlorite treatment for seed sanitation. However, many growers and consumers have become wary of the impact of hypochlorite on human health and the environment and are actively seeking less caustic approaches. Here, we evaluated the effects of both the traditional hypochlorite treatment and a milder alternative on nontyphoidal Salmonella enterica colonization of germinating alfalfa seed. Moreover, we explored three biological factors as potential contributors for inhibition of S. enterica growth: colonization by indigenous bacteria, seed composition changes, and seed metabolite release. In this experimental setting, we found that a combinatorial treatment of heat, peroxide, and acetic acid was as effective as hypochlorite for inhibiting S. enterica growth. Notably, we pinpointed *N*-acetyl-spermidine as an endogenous metabolite exuded by treated seeds that strongly inhibits S. enterica growth. In doing so, we both elucidated one of the mechanisms of chemical sanitation and highlighted a potential seed-derived mode of antimicrobial treatment that may apply to modernized food safety protocols.

**IMPORTANCE** Warm, humid, and nutrient-rich conditions that are used to produce sprouts encourage Salmonella enterica to proliferate. However, many disparate sanitation methods exist, and there is currently no single treatment that can guarantee pathogen-free seeds. Here, we compared the ability of traditional hypochlorite treatment against a combinatorial treatment of heat, peroxide, and vinegar (HPA) commonly used in organic farming practices to inhibit S. enterica colonization and growth during alfalfa germination and found HPA to be at least as effective. Furthermore, we explored seed-based changes following sanitization treatments using metabolomics and identified polyamines as strong inhibitors of *Salmonella* growth on germinating alfalfa. Our findings enable a better understanding of host-pathogen interactions in sprout microbial communities and promote in-depth, evidence-based research in seed sprout safety.

## Abstract (precis)

Warm, humid, and nutrient-rich conditions that are used to produce sprouts encourage Salmonella enterica to proliferate. However, many disparate sanitation methods exist, and there is currently no single treatment that can guarantee pathogen-free seeds.

## INTRODUCTION

Germinated seeds, beans, or grains harvested before the development of true leaves, commonly referred to as sprouts, are consumed in various forms worldwide. Some sprout varieties, notably alfalfa sprouts, are primarily eaten without cooking to preserve optimum flavor, texture, and purported health-promoting properties conferred by bioactive phytochemicals (1, 2). Sprouting seed is commonly germinated at temperatures between 21 and 25°C under constant irrigation to promote optimal seed germination and shoot development. Unfortunately, these conditions are conducive to the growth of bacteria, including pathogenic enterobacterial species that occasionally contaminate seed. Consumption of raw sprouts has been linked to numerous outbreaks of foodborne disease in the past 2 decades and is now recognized as a significant food safety concern (3). The deadliest sprout-associated outbreak reported to date occurred in 2011 in Germany, where fenugreek sprouts served as the vehicle of transmission of a virulent hemorrhagic Escherichia coli strain, resulting in nearly 4,000 infections and 53 deaths (4). Alfalfa sprouts are presently the most commonly reported cause of sprout-associated outbreaks. In the United States alone, 28 outbreaks were reported between 2001 and 2017, resulting in 1,093 illnesses, 110 hospitalizations, and 2 deaths (5). Twenty were associated with alfalfa sprouts contaminated with Salmonella enterica, which can grow exponentially for 3 to 7 days on germinating alfalfa seed (6, 7).

Treatment of seed with an antimicrobial agent to achieve a minimum of 3 log reductions before germination is recommended as a means to minimize microbial hazards in sprouted vegetables (8). The U.S. Food and Drug Administration (FDA) long recommended treatment of sprouting seed with a 20,000-ppm calcium hypochlorite solution prior to germination as a means to reduce microbial contaminants of public health significance and the attendant risk of their growth during germination (9). In response to concerns about worker safety and environmental risks implied by the handling and disposal of concentrated hypochlorite solutions, the FDA issued revised guidance in 2017 to allow the use of any scientifically valid seed sanitation treatment performed in accordance with established safety, recordkeeping, and U.S. Environmental Protection Agency guidelines (10). The revised guidance also provides an opportunity for stakeholders to pursue organic certification, as current organic food production rules prohibit contact of fresh produce with water containing levels of chlorine above drinking water standards (up to 4 ppm) (11).

Several approaches that could be considered for seed sanitation include chemical treatment with calcium hydroxide, acetic acid, lactic acid, or hydrogen peroxide, the application of physical treatments involving heat, high pressure, and radiation, or combinations thereof (10). However, none of the sanitation treatments investigated to date can reliably eliminate all bacterial hazards on sprouting seed or the risk of growth by surviving cells during germination (3, 12). Moreover, such treatments have been shown to influence the composition of bacterial communities that develop on growing sprouts, which are readily colonized by *Pseudomonas* spp., *Lactococcus* spp., *Bacillus* spp., and plant-indigenous *Enterobacteriaceae*, including species (e.g., Pseudomonas fluorescens, Pseudomonas jessenii, and Enterobacter asburiae) antagonistic to enterobacterial pathogens (13–18). Shifts in bacterial community composition provoked by seed sanitation are generally attributed to variable genus- or species-specific susceptibilities to chemical or physical stresses incurred during treatment. However, the extent to which plant host-derived factors shape the development of bacterial communities and the fate of enterobacterial pathogenic species on germinating sprouting seed has not been explored.

Plant tissues exude a range of molecules, including amino acids, sugars, and organic acids, that are utilized directly as growth substrates or as precursors for the biosynthesis of growth-limiting metabolites by colonizing bacteria (19–22). Conversely, molecules with repellant or antibacterial properties, such as phenolics, terpenes, steroids, and alkaloids, fatty acids, and acyl sugars, may also be released, notably under stressful environmental conditions (23, 24). For example, sugars, sugar alcohols, and organic acids have been shown to stimulate the growth of S. enterica in the tomato plant phyllosphere, whereas fatty acids, including palmitic and oleic acids, have the opposite effect (25).

**FIG 1:**
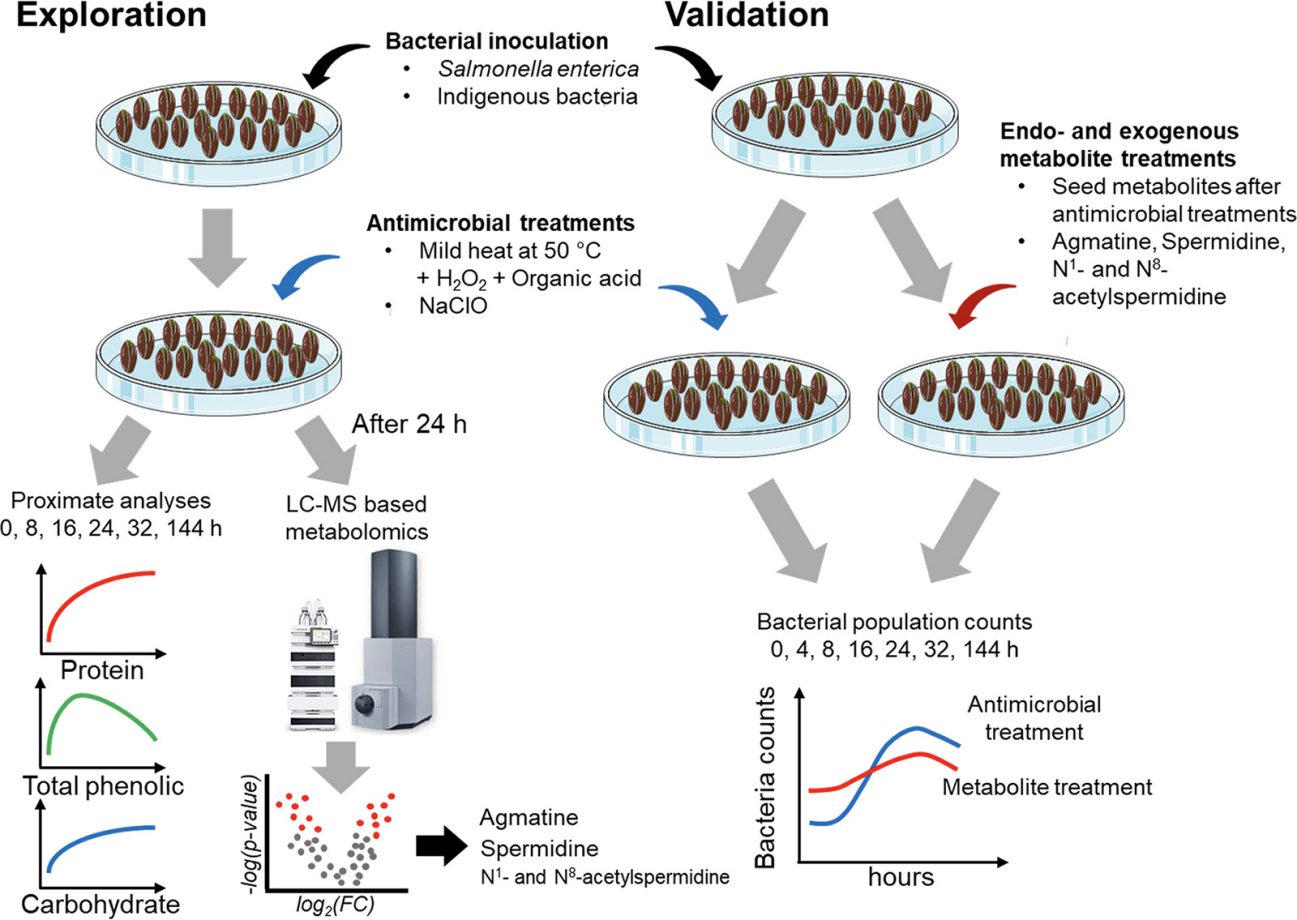
Workflow for this study. (Left) Exploration to identify metabolites from germinating seeds inoculated with S. enterica and investigate change of proximate composition during seed germination. (Right) Validation of effect of seed metabolites on S. enterica growth. Antimicrobial treatments and their abbreviations used in this study include heat plus hydrogen peroxide plus acetic acid (HPA) and sodium hypochlorite (CLO). For study controls, see Materials and Methods for more details.

In the present work, we sought to identify key metabolites released by germinating alfalfa seed that could play an underlying role in inhibiting of *Salmonella* growth following common sanitation treatments, specifically in the context of hypochlorite, as traditionally recommended by regulatory agencies, and hydrogen peroxide-based methods common to organic farming practices. In an exploration phase, we took a multifaceted approach, examining impacts on *Salmonella* colonization of alfalfa seed by indigenous bacteria, as well as changes to seed composition and to the seed metabolome. In a validation phase, we investigated whether the distinct metabolome of treated seeds or specific metabolites of interest could be sufficient to inhibit of *Salmonella* growth if applied to contaminated seeds exogenously. Figure 1 shows our workflow encompassing both stages.

## RESULTS AND DISCUSSION

### Moderate S. enterica growth occurred on sanitized alfalfa seed.

The growth of five S. enterica strains was measured on germinating alfalfa seed subjected to three sanitation treatments, including sodium hypochlorite (CLO) and heat, peroxide, and vinegar (HPA) (Table 1). Treatment with concentrated chlorine or hydrogen peroxide solutions is currently recommended by regulatory agencies, and the HPA treatment represents an alternative approach compliant with organic production principles that relies on multiple “hurdles” to achieve a synergistic antibacterial effect (26). None of the S. enterica strains were detected on alfalfa seed immediately after treatment by the methods used in this study (limit of detection = 1 log CFU/g). In contrast, populations had increased by 5.1 to 7.6 and 4.8 to 6.6 log CFU/g on germinating CLO- and HPA-treated seeds, respectively, after 1 day (Table 1). After 6 days of germination, S. enterica populations reached 4.5 to 7.6 log CFU/g on untreated seed and 6.0 to 7.1 and 3.9 to 6.0 log CFU/g on CLO- and HPA-treated seed (Table 1). Salmonella enterica serovar Agona PARC 5 populations were highest after 1 day of germination (7.58 ± 0.15 log CFU/g), while Salmonella enterica serovar Typhimurium LMFS-S-JF-001 populations were significantly lower (*P < *0.05). Overall, S. enterica populations on germinating seeds subjected to the HPA treatment were consistently lower than those measured on seed treated with CLO. These findings are in line with previous studies that have shown the combinatorial treatment of heat, peroxide and acetic acid to be effective in hindering growth of Escherichia coli O157:H7, Listeria monocytogenes, and S. enterica inoculated onto alfalfa and radish seeds (27).

**TABLE 1:** Populations of five S. enterica strains and total aerobic mesophilic populations on germinating alfalfa seed subjected to three sanitation treatments^*a*^

S. enterica serotype and strain	Treatment	Population (log CFU/g) after days of germination^*b*^					
		0 (initial)		1		6	
		XLD	APC	XLD	APC	XLD	APC
Agona PARC 5	CTL	4.59 ± 0.36 a	3.79 ± 0.34 a	8.96 ± 0.11 a	8.45 ± 0.44 a	7.55 ± 0.12 a	8.97 ± 0.59 a
	CLO	<1 b	3.87 ± 0.37 a	7.58 ± 0.15 b	8.25 ± 0.07 a	7.14 ± 0.32 a	9.29 ± 0.35 a
	HPA	<1 b	4.25 ± 0.81 a	6.24 ± 0.47 c	7.82 ± 0.65 a	5.95 ± 0.30 b	9.53 ± 0.41 a
Agona FSL S5-517	CTL	4.59 ± 0.14 a	5.18 ± 0.62 a	7.85 ± 0.09 a	7.48 ± 0.08 a	6.80 ± 0.21 a	8.02 ± 0.19 a
	CLO	<1 b	5.30 ± 0.71 a	7.04 ± 0.48 ab	7.62 ± 0.07 ab	6.45 ± 0.26 ab	7.89 ± 0.25 a
	HPA	<1 b	4.71 ± 0.97 a	6.31 ± 0.51 bc	7.91 ± 0.16 b	5.82 ± 0.55 b	8.40 ± 0.62 a
Enteritidis LMFS-S-JF-005	CTL	4.67 ± 0.40 a	5.54 ± 0.60 a	7.62 ± 0.22 a	7.80 ± 0.41 a	6.58 ± 0.11 a	7.89 ± 0.63 a
	CLO	<1 b	5.42 ± 0.63 a	7.03 ± 0.50 a	7.55 ± 0.20 a	6.64 ± 0.44 a	7.86 ± 0.47 a
	HPA	<1 b	4.85 ± 0.91 a	6.55 ± 0.42 ab	7.67 ± 0.44 a	6.06 ± 0.96 ab	7.91 ± 0.30 a
Daytona LMFS-S-JF-009	CTL	4.22 ± 0.44 a	5.67 ± 0.25 a	7.52 ± 0.31 a	7.92 ± 0.68 a	6.79 ± 0.62 a	8.25 ± 0.19 a
	CLO	<1 b	5.25 ± 0.91 a	6.13 ± 0.36 b	7.43 ± 0.10 a	6.04 ± 0.46 a	8.23 ± 0.12 a
	HPA	<1 b	4.65 ± 0.69 a	6.05 ± 0.24 b	7.50 ± 0.03 a	5.65 ± 0.18 ab	8.28 ± 0.63 a
Typhimurium LMFS-S-JF-001	CTL	3.41 ± 0.03 a	4.52 ± 0.34 a	7.11 ± 0.81 a	8.31 ± 0.21 a	4.46 ± 0.52 a	9.27 ± 0.39 a
	CLO	<1 b	4.19 ± 0.15 a	5.09 ± 0.27 b	8.25 ± 0.22 a	6.00 ± 1.31 a	9.13 ± 0.29 a
	HPA	<1 b	4.24 ± 0.36 a	4.75 ± 0.28 b	8.19 ± 0.11 a	3.88 ± 0.81 a	9.48 ± 0.02 a

a Seed was germinated in the dark at 22.0 ± 0.5°C for 6 days. Abbreviations: XLD, xylose lysine deoxycholate agar; APC, aerobic plate count; CTL, untreated control; CLO, sodium hypochlorite treatment; HPA, heat-hydrogen peroxide-acetic acid treatment.

b Data are means ± standard deviations. Means followed by the same lowercase letter are not significantly different (*P *≥ 0.05) in a comparison of treatment types within the same day and *Salmonella* strain.

### Cocolonization by indigenous bacteria is affected by S. enterica but not by sanitation.

The fastest increase in mesophilic aerobic bacteria populations occurred on seed inoculated with *S.* Typhimurium, reaching more than 9 log CFU/g by day 6 (Table 1). Overall, the sanitation treatments had less of an effect on indigenous bacterial populations, as detected by aerobic plate counts, than on S. enterica. Antimicrobial treatments did not result in a significant reduction of the population of indigenous aerobic bacteria on alfalfa seed inoculated with S. enterica compared to controls (CTL). These findings are comparable to previous work that showed that populations of indigenous bacteria were not reduced by more than 1 log by treatment with H_2_O_2_, peroxyacetic acid plus H_2_O_2_, acidified NaClO_2_, NaClO_2_, EDTA, Na_3_PO_4_, and NaClO unless they were applied at concentrations that induced phytotoxic effects (28).

Due to the distinct growth characteristics of *S*. Agona PARC 5 (the most rapidly growing among the strains tested) and *S.* Typhimurium LMFS-S-JF-001 (the slowest growing), these two strains were selected for comparison across all remaining analyses.

### The chemical composition of alfalfa seed changes dynamically during germination.

Changes in the chemical composition of alfalfa seed during germination and after sanitation have important implications for seed health (necessary to maintain freshness and germination rate), phenolic antioxidant production (with potentially protective effects for both the seeds and consumers), and nutrient production (supporting sprout growth and impacting developing microbiota).

**FIG 2:**
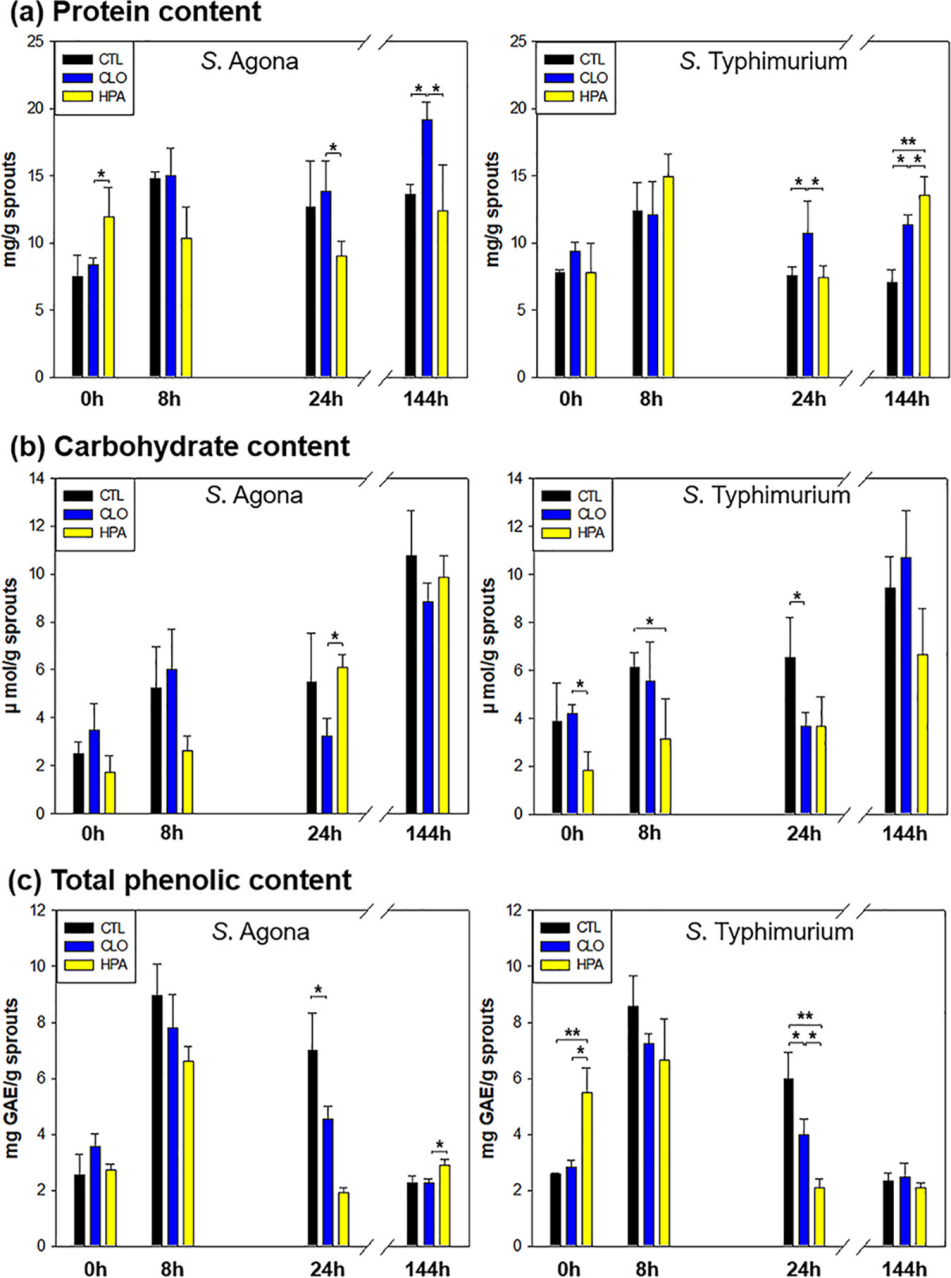
Chemical composition change of germinating alfalfa seed. Protein content (milligrams per gram of sprouts) (a), carbohydrate content (micromoles per gram of sprouts) (b), and total phenolic content (milligrams of gallic acid equivalent [GAE] per gram of sprouts) (c) of germinating alfalfa seeds inoculated with *S.* Agona PARC 5 and *S.* Typhimurium LMFS-S-JF-001 during a 6-day germination period. *, *P* < 0.05; **, *P* < 0.001. Abbreviations: CTL, untreated control; CLO, sodium hypochlorite treatment; HPA, heat-hydrogen peroxide-acetic acid treatment.

Because HOCl and H_2_O_2_ are known to cause protein modification and damage (29, 30), we hypothesized that all three sanitization treatments could affect protein content. However, we did not find significant differences in total protein due to any of the treatments compared to control treatment (Fig. 2a; also, see Table S1 in the supplemental material). Protein content remained relatively constant during germination, increasing slightly from 7 to 12 mg/g immediately after treatment to 7 to 19 mg/g thereafter, irrespective of S. enterica strain. Soluble carbohydrate content also increased during germination from 1.7 to 4.2 μmol/g at the outset to 5.5 to 10.8 μmol/g after 6 days (Fig. 2b; Table S1). In line with these findings, Ling and Chang (31) reported that total soluble carbohydrate contents in red beans and guava seeds continuously increase during germination. Accumulation of carbohydrates can be ascribed to continued hydrolysis of 1,6-galactosidic linkages of glycoproteins, glycolipids, and polysaccharides catalyzed by α-galactosidase (32).

10.1128/mSystems.00898-20.1 TABLE S1 Protein contents (milligrams per gram of sprouts), total phenolic contents (milligrams of gallic acid equivalent per gram of sprouts), and carbohydrate contents (micromoles per gram of sprouts) of sprouting alfalfa seeds inoculated with *S.* Agona PARC 5 and *S*. Typhimurium LMFS-S-JF-001 during the 6-day germination period. Download Table S1, DOCX file, 0.02 MB. Copyright © 2021 Ban et al. 2021 Ban et al. This content is distributed under the terms of the Creative Commons Attribution 4.0 International license.

Interestingly, total phenolic contents increased dramatically during the first 8 h of germination, followed by a gradual decrease between 8 and 24 h and stabilization after 24 to 32 h (Fig. 2c; Table S1). Untreated seed inoculated with either of the S. enterica strains contained the highest levels of total phenolics after 8 to 24 h. This is consistent with previous studies that showed that sprouting seeds tend to synthesize phenols during imbibition and early embryonic development (1) to support cell wall, hormone, and other plant regulator synthesis (33). After 6 days, we found that levels of phenolics were substantially lower, irrespective of treatment, ranging from 1.9 to 2.9 mg gallic acid equivalents (GAE)/g sprouts. This likely reflects the previously observed trend for phenolics to steadily decrease between 24 h and 8 days after initiation of germination (34). High phenolic concentrations in plants used for food and medicine have been correlated with antimicrobial effects (35); however, we did not clearly observe this phenomenon here. For example, while phenolic counts in HPA-treated seeds were much lower than those in CLO-treated seeds from 24 h to 6 days, we detected similar increases in colonization, as measured by aerobic plate count (APC), in both settings.

### Sanitation strongly affects metabolite profiles in germinating alfalfa seed.

**FIG 3:**
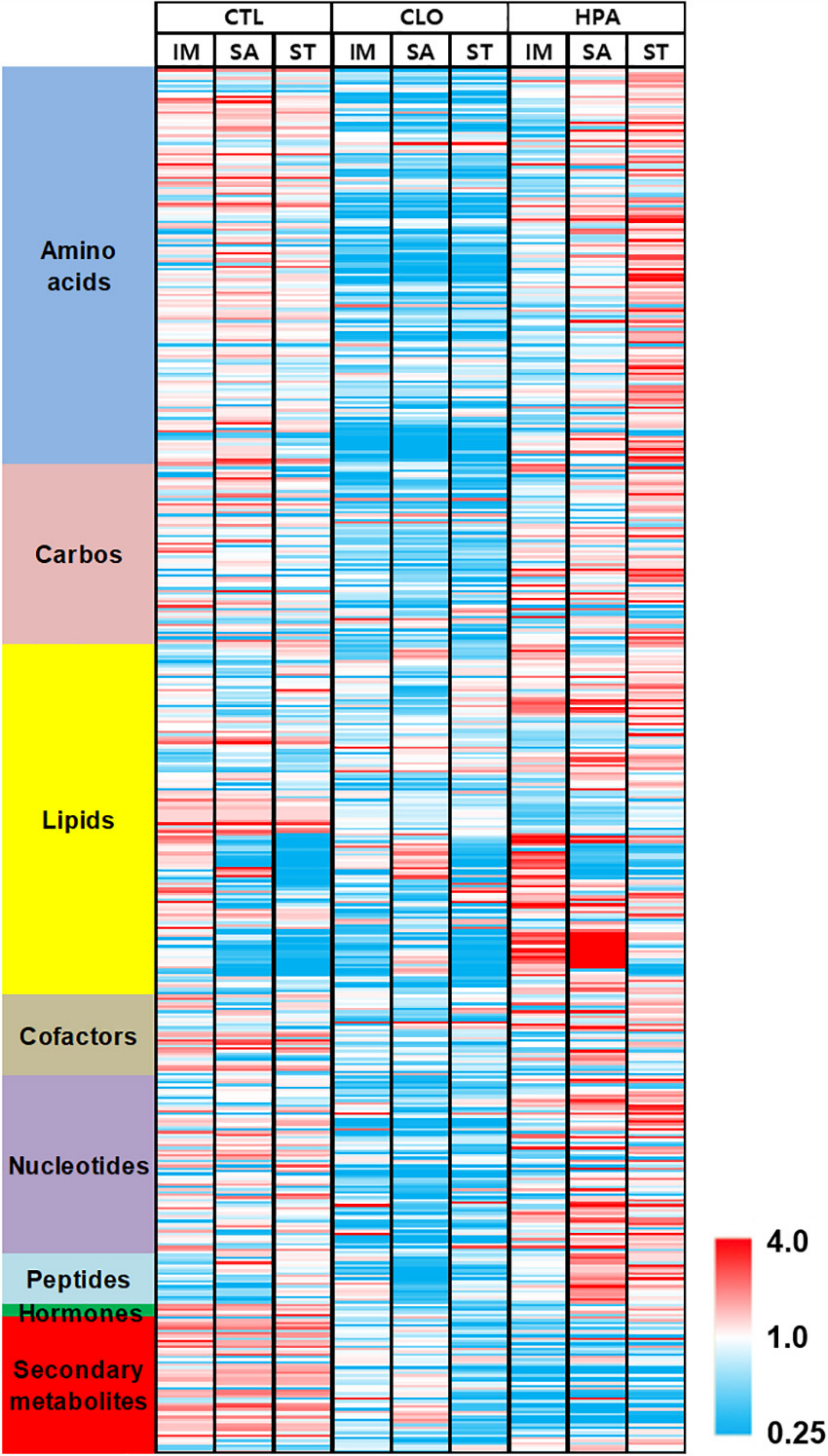
Heat map representation of metabolite scaled intensities in samples grouped by major metabolite class. Log-transformed metabolite concentrations were scaled to the median value (1.0) of all samples for each compound and are represented as different colors (red or blue) with different color intensity. A continuously increasing intensity of red represents values ranging from 1.0 to 4.0, and blue represents values from 1.0 to 0.25. The maximum-intensity red represents all values of ≥4.0, and maximum blue intensity represents values of ≤0.25. The summary of data from four biological replicates is shown for each treatment and inoculation group. Abbreviations: CTL, untreated control; CLO, sodium hypochlorite treatment; HPA, heat - hydrogen peroxide - acetic acid treatment; IM, indigenous microbiota; SA, *S*. Agona PARC 5; ST, *S.* Typhimurium LMFS-S-JF-001.

Metabolomic analysis was performed on exudates from germinating alfalfa seed inoculated with either *S.* Agona or *S.* Typhimurium subjected to two sanitation treatments. A heat map representation of metabolite intensities (Fig. 3) visually demonstrates the effect of treatment type on the abundance of 96.1% of the 535 identified metabolites, which spanned almost all major metabolic pathways, as well as the subtler influence of colonizing S. enterica strain. Principal-component analysis (PCA) of the metabolite profiles enabled elimination of uninformative background variables and visualization of small profile changes. The PCA plot was used to reveal information regarding differences among surface metabolite profile of germinating alfalfa seed and to assess which variables of antimicrobial treatments mostly contributed to this difference (Fig. S1). Principal component 1 (PC1) and PC2 of indigenous bacteria, *S*. Agona, and *S.* Typhimurium LMFS-S-JF-001, explained 69.8 and 13.6%, 53.9 and 25.5%, and 67.5 and 17.2% of the total variability of the data, accounting for the metabolite composition changes in germinating alfalfa seed. The plot indicated that distinct segregation and clustering were apparent on HPA-treated S. enterica.

10.1128/mSystems.00898-20.9 FIG S1 Principal-component analysis (PCA) of the metabolite profiles. PCA of metabolites in germinating alfalfa seed with indigenous microbiota (a), *S*. Agona PARC 5 (b), and *S*. Typhimurium LMFS-S-JF-001 (c) after three different sanitization treatments. Abbreviations: CTL, untreated control; CLO, sodium hypochlorite treatment; HPA, heat-hydrogen peroxide-acetic acid treatment. Download FIG S1, PDF file, 0.2 MB. Copyright © 2021 Ban et al. 2021 Ban et al. This content is distributed under the terms of the Creative Commons Attribution 4.0 International license.

Fold changes (FC) of the metabolites were log transformed as 1 log of FC. All values were reported for completeness, and independent *t* tests were applied to define significant increase or decrease. Compounds that were significantly increased or decreased (*P < *0.05) by both the CLO and HPA treatments included a wide range of metabolites from all biochemical classes, especially amino acids and secondary metabolites (Table S2). Compounds that were significantly increased (*P < *0.05) by both treatments relative to the control included some carbohydrates related to oxidation products of larger biochemical compounds (e.g., oxalate) and breakdown products of cell walls or larger oligosaccharides (e.g., fucitol, maltose, and verbascose). Several markers for protein, membrane, and nucleic acid degradation (e.g., dimethylarginine) were significantly increased following HPA treatment but decreased following CLO treatment (*P < *0.05). Levels of 25 amino acids were significantly increased upon colonization of both *S*. Agona PARC 5 and *S.* Typhimurium LMFS-S-JF-001 after the HPA treatment, while no amino acids were significantly decreased (*P < *0.05) (Table S3). In addition, the concentrations of 10 phospholipids were significantly decreased in both HPA-treated S. enterica-colonized samples (*P < *0.05) (Table S4). In this study, due to the vast bulk of the sample used for metabolite analysis, which was composed of seed exudate material (estimated 99.5% of sample volume, as opposed the indigenous and pathogenic bacteria contributing roughly 0.5% of sample volume), we attributed the levels of metabolites mainly to seeds. A caveat here is that we cannot completely rule out the possibility that some metabolites produced by bacteria could also contribute to expression changes. However, the fact that we did not detect any amino sugars in our analysis (a metabolite commonly produced by bacteria but not synthesized in any significant amounts by plants) (36) seems to point to the fact that our results are representative of metabolites derived from seed and not the colonizing bacteria.

10.1128/mSystems.00898-20.2 TABLE S2 Metabolites significantly increased and decreased on germinating alfalfa seed treated with CLO and HPA treatments compared to CTL at 24 h of germination (*P < *0.05). Download Table S2, DOCX file, 0.03 MB. Copyright © 2021 Ban et al. 2021 Ban et al. This content is distributed under the terms of the Creative Commons Attribution 4.0 International license.

10.1128/mSystems.00898-20.3 TABLE S3 Fold-change increase and decrease in amino acids expression in germinating alfalfa seed inoculated with SA or ST after HPA treatment at 24 h of germination compared to control (0 h). Download Table S3, DOCX file, 0.02 MB. Copyright © 2021 Ban et al. 2021 Ban et al. This content is distributed under the terms of the Creative Commons Attribution 4.0 International license.

10.1128/mSystems.00898-20.4 TABLE S4 Fold-change decrease in phospholipid expression in germinating alfalfa seed inoculated with *S.* Agona or *S.* Typhimurium after HPA treatment at 24 h of germination compared to controls (0 h). Download Table S4, DOCX file, 0.02 MB. Copyright © 2021 Ban et al. 2021 Ban et al. This content is distributed under the terms of the Creative Commons Attribution 4.0 International license.

### Germinating seed exudates have moderate antimicrobial effects against S. enterica.

**FIG 4:**
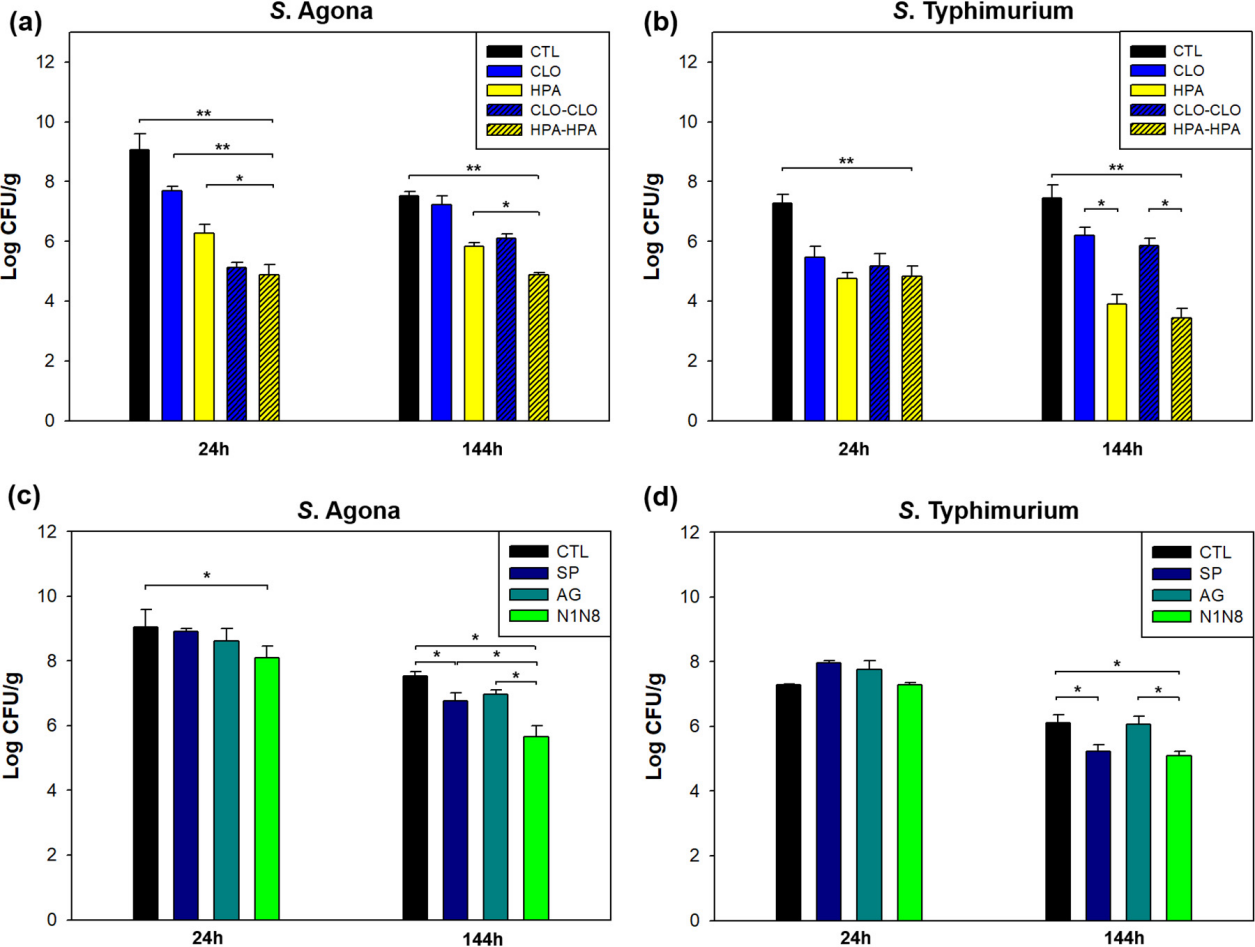
Metabolite effects on S. enterica growth. Populations of *S*. Agona PARC 5 (a and c) and *S.* Typhimurium LMFS-S-JF-001 (b and d) in germinating alfalfa seed after supplementation with metabolites from CLO-CLO (injection of metabolites from CLO-treated alfalfa seeds on CLO-treated alfalfa seeds inoculated with S. enterica) or HPA-HPA (injection of metabolites from HPA-treated alfalfa seeds on HPA-treated alfalfa seeds inoculated with S. enterica) and spermidine, agmatine, and *N*^1^- and *N*^8^-acetylspermidine. *, *P* < 0.05; **, *P* < 0.001. Abbreviations: CTL, untreated control; CLO, sodium hypochlorite treatment; HPA, heat-hydrogen peroxide-acetic acid treatment.

Seed exudates harvested from CLO- or HPA-treated seeds after 24 h of germination were applied to CLO- or HPA-treated seeds inoculated with S. enterica to determine whether metabolites released by the treatments affected colonization. The variable effects of the treatments are shown in Fig. 4a and b (see also Tables S5 and S6). HPA-treated sprouts injected daily with exudates from HPA-treated seeds exhibited the most significant antimicrobial effects (*P < *0.001) up to 2.4 log CFU/g for *S*. Agona PARC 5 and 2.7 log CFU/g for *S.* Typhimurium LMFS-S-JF-001 compared to HPA-treated sprouts with no additional injection of exudates. One caveat to these findings is that although seed exudates were filtered before application, residual indigenous bacteria could still have affected microbiomes and secondary metabolites of inoculated seeds (37). Such interactions between indigenous bacterial inoculants and plant microbial communities, in addition to changes in plant production of bioactive metabolites, have been reported (38).

10.1128/mSystems.00898-20.5 TABLE S5 Populations (log CFU per gram) of *S.* Agona PARC 5 on germinating CLO- or HPA-treated alfalfa seed after injection with an extract from CLO- or HPA-treated alfalfa sprouts. Download Table S5, PDF file, 0.1 MB. Copyright © 2021 Ban et al. 2021 Ban et al. This content is distributed under the terms of the Creative Commons Attribution 4.0 International license.

10.1128/mSystems.00898-20.6 TABLE S6 Populations (log CFU per gram) of *S.* Typhimurium LMFS-S-JF-001 on germinating CLO- or HPA-treated alfalfa seed after inoculation with an extract from CLO- or HPA-treated alfalfa sprouts. Download Table S6, PDF file, 0.1 MB. Copyright © 2021 Ban et al. 2021 Ban et al. This content is distributed under the terms of the Creative Commons Attribution 4.0 International license.

### Polyamines significantly reduce S. enterica growth on germinating alfalfa seed.

The polyamines *N*-acetylputrescine and *N*^1^- and *N*^8^-acetylspermidine were significantly increased (*P < *0.05) on control and CLO-treated germinating seed inoculated with S. enterica compared to control and CLO-treated germinating seed with indigenous bacteria (data not shown). We therefore examined the putative antimicrobial effects of the polyamines spermidine, agmatine, and N^1^- and N^8^-acetylspermidine on the growth of both *S*. Agona and *S.* Typhimurium (Fig. 4c and d; Tables S7 and S8). Strikingly, populations of both *S*. Agona and *S.* Typhimurium decreased significantly after 24 and 48 h, respectively, particularly on seed injected with *N*^1^- and *N*^8^-acetylspermidine (*P < *0.05) despite substantial initial population increases of 8.0 to 8.91 log CFU/g (*S*. Agona PARC 5) and 7.47 to 8.27 log CFU/g (*S.* Typhimurium LMFS-S-JF-001) during the first day of germination (Tables S7 and S8). Effects may have been more apparent at later time points (after 24 h) due to repeated daily polyamine injections. In a previous study, the application of 2 to 4 mM exogenous spermine was found be sufficient to inhibit bacterial growth on isolated bacterial cultures of *S.* Typhimurium LT2 and E. coli K-10 (39). Our study builds upon this prior work by demonstrating similar inhibitory effects at similar concentrations *in vivo* on germinating alfalfa seed. Specifically, our exogenous applications of 500 ppm acetylspermidine were equivalent to 2.7 mM, and while the concentration of endogenous spermidine in the alfalfa exudates was somewhat lower at 1.2 mM, antimicrobial effects were still observed.

10.1128/mSystems.00898-20.7 TABLE S7 Populations (log CFU per gram) of *S.* Agona PARC 5 on germinating alfalfa sprouts supplemented with spermidine, agmatine, and *N*^1^- and *N*^8^-acetylspermidine. Download Table S7, PDF file, 0.1 MB. Copyright © 2021 Ban et al. 2021 Ban et al. This content is distributed under the terms of the Creative Commons Attribution 4.0 International license.

10.1128/mSystems.00898-20.8 TABLE S8 Populations (log CFU per gram) of *S.* Typhimurium LMFS-S-JF-001 on germinating alfalfa sprouts supplemented with spermidine, agmatine, and *N*^1^- and *N*^8^-acetylspermidine. Download Table S8, PDF file, 0.1 MB. Copyright © 2021 Ban et al. 2021 Ban et al. This content is distributed under the terms of the Creative Commons Attribution 4.0 International license.

In addition, data on the growth of *S*. Agona PARC 5 and *S.* Typhimurium LMFS-S-JF-001 on germinating alfalfa seed supplemented with polyamines were suggestive of longer lag phases leading to delays in the initiation of exponential growth (Table 2). Taken together, these observations indicate that *N*^1^- and *N*^8^-acetylspermidine and other polyamines contribute antimicrobial effects during the germination of seed treated with hypochlorite. However, spermidine has been reported to cause acute toxicity at 600 mg/kg body weight and the no-observed-adverse-effect level of 1,000 ppm (83 mg/kg body weight/day) (40). For this reason, the extent to which polyamines can be applied to germinating seeds without toxicity, as well as negative effects impacting sprout palatability, nutritional content, storage, germination, and productivity for growers, remains to be seen.

**TABLE 2:** Growth kinetic parameters of *Salmonella* strains on germinating seeds supplemented with spermidine, agmatine, and *N*^1^- and *N*^8^-acetylspermidine^*a*^

Supplement	Concn (ppm)	*S*. Agona PARC 5			*S*. Typhimurium LMFS-S-JF-001		
		Lag time (h)	Max rate (h^−1^)	Final value	Lag time (h)	Max rate (h^−1^)	Final value
Control	0	3.46 ± 0.16 a	0.12 ± 0.01 ab	1.02 ± 0.01 a	4.22 ± 0.13 d	0.16 ± 0.01 a	0.83 ± 0.01 a
Spermidine	10	3.59 ± 0.37 ab	0.09 ± 0.02 bc	0.90 ± 0.13 a	3.99 ± 0.25 de	0.10 ± 0.04 d	0.85 ± 0.04 a
	100	3.63 ± 0.37 ab	0.09 ± 0.02 bc	0.94 ± 0.10 a	4.90 ± 0.21 b	0.12 ± 0.01 c	0.87 ± 0.02 a
	500	4.11 ± 0.28 cd	0.10 ± 0.02 abc	1.01 ± 0.16 a	5.21 ± 0.22 ab	0.11 ± 0.01 bc	0.89 ± 0.05 a
Agmatine	10	3.74 ± 0.17 abc	0.08 ± 0.02 c	0.89 ± 0.15 a	3.91 ± 0.18 e	0.09 ± 0.04 bc	0.84 ± 0.03 a
	100	3.82 ± 0.28 bcd	0.09 ± 0.03 bc	0.97 ± 0.09 a	4.90 ± 0.07 b	0.10 ± 0.00 bc	0.77 ± 0.05 a
	500	3.93 ± 0.23 bcd	0.09 ± 0.02 abc	0.92 ± 0.10 a	5.03 ± 0.13 b	0.10 ± 0.03 bc	0.82 ± 0.02 a
*N*^1^- and *N*^8^-acetylspermidine	10	3.77 ± 0.21 abc	0.10 ± 0.01 abc	0.91 ± 0.05 a	4.58 ± 0.25 c	0.09 ± 0.04 bc	0.83 ± 0.05 a
	100	4.21 ± 0.12 d	0.12 ± 0.02 a	0.88 ± 0.05 a	5.00 ± 0.17 b	0.11 ± 0.05 b	0.82 ± 0.10 a
	500	4.65 ± 0.26 e	0.10 ± 0.01 abc	0.96 ± 0.12 a	5.45 ± 0.15 a	0.11 ± 0.01 bc	0.83 ± 0.03 a

a Data are means ± standard deviations. Means followed by the same lowercase letter in the same column are not significantly different (*P *≥ 0.05).

Until effective and reliable treatments can be developed, multihurdle approaches and microbial testing of spent irrigation water remain of utmost importance to ensure that contaminated sprouts do not enter the marketplace. Although we find that the regrowth of S. enterica in sprouts following sanitization treatments is strain dependent and treatment dependent, the mechanisms responsible for this effect require further investigation. Moreover, deeper studies of the microbiomes in alfalfa seed could be necessary to enhance our understanding of the role of microbiota in germinating alfalfa sprouts. The results of this study provide new insights on mechanisms that influence interactions between enterobacterial pathogens and germinating sprouting seed. With better understanding of the interactions between S. enterica and indigenous bacteria and sprouting of seeds, more targeted interventions can be developed for the production of pathogen-free sprouted vegetables.

## MATERIALS AND METHODS

### Bacterial strains and culture conditions.

Five strains from four serotypes of S. enterica, including S. enterica serotype Agona (PARC 5 and FSL S5-517), S. enterica serotype Enteritidis (LMFS-S-JF-005), S. enterica serotype Daytona (LMFS-S-JF-009), and S. enterica serotype Typhimurium (LMFS-S-JF-001), were used in this study. The strains were stored at −80°C in brain heart infusion (BHI) broth (Becton Dickinson [BD], New Jersey, USA) supplemented with 20 to 25% glycerol. Working stocks were maintained on tryptic soy agar (TSA; BD Canada, Ontario, Canada) at 4°C for a maximum of 1 month.

### Alfalfa seeds and seed inoculation.

Certified organic alfalfa sprouting seed was obtained from a Canadian seed producer (Mumm’s Sprouting Seeds, Saskatchewan, Canada). A single colony of each S. enterica strain was transferred to 10 ml tryptic soy broth (TSB) (BD Canada, Ontario, Canada) and incubated at 37°C for 18 ± 0.25 h with agitation to achieve a cell density of approximately 8 log CFU/ml. The culture was then spun in a centrifuge at 3,000 rpm for 10 min at 22°C, the supernatant was discarded, and the pellet was resuspended in 10 ml of a 0.85% saline solution. Subsequently, 500-μl aliquots from each culture were added to 9.5 ml 0.85% saline, and 10 ml of the diluted culture was added to 100.0 ± 0.1 g alfalfa seeds in 500-ml sterile Schott bottles. The bottles were rolled for 1 min to distribute the inoculum through the seed mass, and the contents were transferred to sterile paper sheets in a biosafety cabinet, where they were held for 2 h.

### Antimicrobial seed treatments and germination of alfalfa seed.

Alfalfa seed inoculated with each S. enterica strain was subjected to three antimicrobial treatments: immersion in a 5,000-ppm sodium hypochlorite solution (NaClO) (Ricca Chemical Company, Texas, USA) for 20 min (CLO) and a treatment compliant with organic production practices (HPA) consisting of immersion in water heated to 50°C for 10 min followed by immersion in 2% hydrogen peroxide (BDH, Alberta, Canada) with 0.1% acetic acid (white vinegar; Heinz Company, Illinois, USA) for 10 min. The concentration of 5,000 ppm NaClO was chosen because 20,000 ppm calcium hypochlorite as recommended by FDA (9) is caustic and can be a potential health hazard to workers who are handling the agent. On the other hand, the Canadian recommendation of 2,000 ppm calcium hypochlorite was not sufficient to kill S. enterica on alfalfa seed in previous studies (27).

Twenty-five grams of alfalfa seed in sterile 500-ml glass bottles was treated with 125 ml of each solution or sterile water (control [CTL]). The bottles were shaken vigorously every 2 min for 10 s. After treatment, the seeds were filtered through Whatman no. 4 filter paper (20 μm) to remove the antimicrobial solution and were rinsed with 125 ml sterile distilled water (DW). Immediately after seed treatment, 20.00 ± 0.05 g treated or untreated seeds was placed in a sterile pipette tip box with a sterile gauze pad moistened with 20 ml sterile DW. The boxes were closed and placed in a darkened incubator set at 22.0 ± 0.5°C for 6 days. Sterile DW (5 ml) was added to each box 24 h after initiation of germination and every day thereafter.

### Enumeration of S. enterica and aerobic mesophilic bacteria on germinating alfalfa seed.

S. enterica populations were estimated from colony counts obtained on the selective medium xylose lysine deoxycholate agar (XLD; BD Canada, ON, Canada). Total aerobic mesophilic populations were estimated on tryptic soy agar (TSA). Seed from each box was sampled at 0 h (immediately after treatment) and after 1 and 6 days of germination. At each sampling interval, 3 g of seed was placed in a 50-ml sterile centrifuge tube with 27 ml phosphate-buffered saline (PBS) (Amresco Inc., Ohio, USA). After mixing on a vortex mixer for 15 s, serial dilutions of the supernatant were prepared in PBS, and 50 μl or 1 ml of the diluted samples was applied to XLD, in duplicate, using a spiral plater (easySpiral Pro; Interscience Laboratories, Inc., Massachusetts, USA). The plates were incubated at 37°C for 24 ± 2 h. Additionally, 50-μl aliquots were applied to TSA using the spiral plater for estimation of aerobic plate count (APC). The TSA agar plates were incubated at 37°C for 24 ± 2 h. Indigenous mesophilic aerobic populations were calculated by subtraction of S. enterica colony counts on XLD agar from colony counts on TSA agar.

### Lyophilization of sprout exudates.

Germinated seeds were collected after 0, 8, 16, 24, and 32 h and 6 days for proximate analyses and 24 h for metabolomics analysis. Samples (10 g) were placed in sterile filtered stomacher bags (Filtra-Bag; VWR, Alberta, Canada) with 10 ml sterile DW and were gently massaged by hand for 2 min. The filtrates were transferred to 100-ml flat-bottom specimen containers (VWR, Alberta, Canada) with 8 ml 100% ethanol and were stored at −20°C for 24 h. The containers were then placed in a high-vacuum chamber for 6 h to remove ethanol, and the residual materials were frozen at −80°C overnight in preparation for lyophilization in a bulk tray freeze-dryer (Labconco, Missouri, USA).

### Proximate analyses.

Germinating seed lyophilizates were dissolved in 1 ml sterile DW for proximate analysis. Protein content was determined by Bradford assay (41); total phenolic content was determined using a Folin-Ciocalteu assay with minor modifications as described by Chen et al. (42); total soluble carbohydrate content was determined by phenol-sulfuric acid methods (43).

### Metabolic profiling.

Sample extraction and preparation were conducted at Metabolon, Inc. (Research Triangle Park, NC, USA), with their mass spectrometer platforms and instrument settings and conditions, as described previously in detail (44, 45) with a few modifications. The major components of the process are summarized as follows. The sample preparation process was carried out using an automated MicroLab STAR system (Hamilton, Nevada, USA). Recovery standards were added prior to the first step in the extraction process for quality control purposes. Sample preparation was conducted using a proprietary series of organic and aqueous extractions to remove the protein fraction while allowing maximum recovery of small molecules. The resulting extract was divided into equal fractions for analysis on three independent platforms: ultrahigh-performance liquid chromatography-tandem mass spectrometry (UHPLC-MS/MS2) optimized for basic molecules (LC/MS-NEG), UHPLC/MS/MS2 optimized for acidic molecules (LC/MS-POS), and UHPLC/MS/MS2 optimized for polar molecules (LC/MS-Polar).

The mass spectrometry data files were extracted, and ion peaks were identified and integrated using Metabolon’s proprietary software. Metabolites were then identified by automated comparison of the ion features in the experimental samples to an in-house reference library of chemical standard entries that included retention time, molecular weight (*m/z*), preferred adducts, and in-source fragments as well as associated MS spectra and curated by visual inspection for quality control using software developed at Metabolon. The reference library, including known and novel metabolites, was developed by analyzing more than 3,000 pure reference standards of known metabolite structures with the identical LC/MS methods and cataloging all the ions that were produced.

### Validation of the effects of germinating seed metabolites on S. enterica growth.

The effects of germinating seed metabolites on S. enterica growth were evaluated by two methods: first, using complete leachates from CLO- or HPA-treated alfalfa seeds, encompassing all seed metabolites but filtered to remove bacterial contamination; second, using specific metabolites of interest, specifically, two polyamines previously implicated in pathogenic virulence (46) and found in this study to be significantly increased in CTL and CLO-treated alfalfa seeds inoculated with S. enterica (*P < *0.05).

To evaluate the effect of metabolites using complete leachates, 25 g of uninoculated seed was rinsed with CLO or HPA over Whatman no. 4 filter paper and germinated for 24 h. The germinated seed was transferred to sterile filter stomacher bags with 10 ml sterile DW, which were gently massaged by hand for 2 min, and passed through a 0.2-μm syringe filter (VWR, Canada) to prepare leachate. Alfalfa seed inoculated with *S*. Agona PARC 5 or *S.* Typhimurium LMFS-S-JF-001 was concomitantly treated with CLO or HPA and were transferred to pipette tip boxes along with 2 ml of leachate. During seed incubation, fresh leachate was injected daily. As a CTL, inoculated alfalfa seed in a pipette tip box was treated with 2 ml of sterile water. Populations of *S*. Agona PARC 5 and *S*, Typhimurium LMFS-S-JF-001 on germinating seeds were enumerated immediately after addition of the leachate and after 4, 8, 12, and 24 h and 6 days.

To evaluate the effect of specific metabolites of interest, 20 ml of polyamine solutions containing either spermidine (Alfa Aesar, Massachusetts, USA), agmatine (TCI America, Oregon, USA), or *N*^1^- and *N*^8^-acetylspermidine (Musechem, New Jersey, USA) at concentrations of 10, 100, and 500 ppm was added daily to 20 g alfalfa seed inoculated with *S.* Agona PARC 5 or *S.* Typhimurium LMFS-S-JF-001 (a range of 10 to 500 ppm was found to have the largest effect on S. enterica growth in preliminary analyses; data not shown). Twenty grams of alfalfa seed in sterile 500-ml glass bottles was treated with 125 ml of each solution or sterile water (CTL). Populations of each S. enterica strain were determined after 0, 4, 8, 12, 24, and 48 h and 6 days of germination by the procedure described above. Growth curves drawn from the data were fitted to the Baranyi and Roberts predictive model using DMfit (v3.5) available on the ComBase browser (http://browser.combase.cc/DMFit.aspx), and the growth kinetic parameters were recorded (47).

### Statistical analysis.

All experiments were repeated at least three times using independently prepared samples. Bacterial populations during germination, protein content, total phenolic content, and soluble carbohydrate content were examined by one-way analysis of variance (ANOVA) followed by Tukey’s honestly significant difference (HSD) test (α = 0.05) using Minitab 17 (Minitab, Inc., Pennsylvania, USA). *P* values of <0.05 were considered statistically significant.

Statistical comparisons of metabolites derived from metabolomics analyses were performed by independent *t* tests using Minitab 17. An estimate of the false discovery rate (*q* value) was calculated to take into account the multiple comparisons that normally occur in metabolomics-based studies. Principal-component analysis (PCA) and all other statistical analyses were performed using ArrayStudio (Omicsoft, North Carolina, USA) on natural log-transformed scaled imputed data.
