# Supplementary material for: Endogenous Metabolites Released by Sanitized Sprouting Alfalfa Seed Inhibit the Growth of Salmonella enterica
Source: mSystems. 2021 Feb 9;6(1):e00898-20. doi: 10.1128/mSystems.00898-20 (PMC7883538; doi:10.1128/mSystems.00898-20)
Supplement: TABLE S1 [file mSystems.00898-20-st001.docx]

**Table S1**

| **Treatments** | ***S. enterica* strains** | **Hours of germination** | | | | | |
| --- | --- | --- | --- | --- | --- | --- | --- |
|  |  | **0** | **8** | **16** | **24** | **32** | **144 (6 days)** |
| **(A) Protein (mg/g sprouts)** | |  |  |  |  |  |  |
| CTL | SA | 7.48 ± 1.62^aB^ | 14.80 ± 0.53^aA^ | 11.74 ± 2.14^aAB^ | 12.69 ± 3.41^aA^ | 11.45 ± 0.83^aAB^ | 13.67 ± 0.68^bA^ |
|  | ST | 7.77 ± 0.24^aB^ | 12.39 ± 2.15^aA^ | 10.95 ± 2.11^aAB^ | 7.60 ± 0.60^aB^ | 9.66 ± 1.56^aAB^ | 7.10 ± 0.94^aB^ |
| CLO | SA | 8.36 ± 0.51^aB^ | 15.02 ± 2.08^aA^ | 14.61 ± 3.07^bAB^ | 13.87 ± 2.23^aAB^ | 14.92 ± 3.54^aA^ | 19.16 ± 1.35^bA^ |
|  | ST | 9.42 ± 0.62^aA^ | 12.08 ± 2.48^aA^ | 8.47 ± 1.61^aA^ | 10.7 ± 2.41^aA^ | 9.00 ± 2.29^aA^ | 11.36 ± 0.76^aA^ |
| HPA | SA | 11.98 ± 2.14^aA^ | 10.35 ± 2.33^aA^ | 10.73 ± 1.73^aA^ | 9.01 ± 1.11^aA^ | 12.14 ± 1.97^bA^ | 12.37 ± 3.43^aA^ |
|  | ST | 7.82 ± 2.13^aB^ | 14.94 ± 1.71^aA^ | 13.84 ± 2.18^aA^ | 7.47 ± 0.83^aB^ | 7.42 ± 0.95^aB^ | 13.57 ± 1.40^aA^ |
| **(B) Carbohydrate (μmol/g sprouts)** | | | | |  |  |  |
| CTL | SA | 2.50 ± 0.48^aC^ | 5.25 ± 1.70^aBC^ | 6.28 ± 0.53^bBC^ | 5.47 ± 2.06^aBC^ | 7.59 ± 1.50^aAB^ | 10.77 ± 1.90^aA^ |
|  | ST | 3.89 ± 1.57^aC^ | 6.14 ± 0.60^aABC^ | 7.28 ± 0.24^aAB^ | 6.52 ± 1.68^aABC^ | 5.77 ± 1.33^aBC^ | 9.45 ± 1.30^aA^ |
| CLO | SA | 3.48 ± 1.11^aB^ | 6.03 ± 1.68^aAB^ | 4.02 ± 1.76^aB^ | 3.25 ± 0.71^aAB^ | 5.05 ± 0.12^aB^ | 8.85 ± 0.76^aA^ |
|  | ST | 4.22 ± 0.37^aB^ | 5.54 ± 1.66^aB^ | 3.86 ± 0.65^aB^ | 3.66 ± 0.59^aB^ | 4.72 ± 1.18^aB^ | 10.72 ± 1.93^aA^ |
| HPA | SA | 1.71 ± 0.71^aC^ | 2.62 ± 0.61^aC^ | 2.52 ± 0.67^aC^ | 6.10 ± 0.52^aB^ | 6.37 ± 0.46^aB^ | 9.87 ± 0.92^aA^ |
|  | ST | 1.84 ± 0.79^aB^ | 3.14 ± 1.66^aAB^ | 2.87 ± 1.22^aB^ | 3.66 ± 1.23^bAB^ | 4.13 ± 1.05^bAB^ | 6.65 ± 1.91^aA^ |
| **(C) Total phenolic (mg gallic acid equivalent/ g sprouts)** | | | |  |  |  |  |
| CTL | SA | 2.56 ± 0.74^aB^ | 8.97 ± 1.10^aA^ | 7.60 ± 1.15^aA^ | 6.99 ± 1.33^aA^ | 2.99 ± 0.74^bB^ | 2.26 ± 0.25^aB^ |
|  | ST | 2.58 ± 0.06^aC^ | 8.56 ± 1.10^aA^ | 8.12 ± 0.55^aA^ | 5.99 ± 0.95^aB^ | 5.32 ± 1.05^aB^ | 2.34 ± 0.27^aC^ |
| CLO | SA | 3.58 ± 0.46^aC^ | 7.81 ± 1.20^aA^ | 6.16 ± 1.80^aAB^ | 4.55 ± 0.47^aBC^ | 2.92 ± 0.29^aC^ | 2.27 ± 0.14^aC^ |
|  | ST | 2.83 ± 0.24^aCD^ | 7.26 ± 0.35^aA^ | 4.66 ± 0.54^aB^ | 3.98 ± 0.56^aBC^ | 2.84 ± 0.35^aCD^ | 2.48 ± 0.48^aD^ |
| HPA | SA | 2.73 ± 0.20^bB^ | 6.61 ± 0.52^aA^ | 5.18 ± 1.53^aA^ | 1.94 ± 0.14^aB^ | 2.03 ± 0.55^aB^ | 2.89 ± 0.23^aB^ |
|  | ST | 5.48 ± 0.89^aAB^ | 6.65 ± 1.46^aA^ | 4.42 ± 0.83^aBC^ | 2.11 ± 0.32^aD^ | 2.39 ± 0.34^aCD^ | 2.10 ± 0.17^bCD^ |

Means labelled with the same lowercase letter were not statistically different when comparing between Agona and Typhimurium within the same treatment time and type of content examined (P ≥ 0.05). Means labelled with the same uppercase letter in the same row are not statistically different when comparing across treatment time points (P ≥ 0.05). Abbreviations: CTL; no treated sample, CLO, sodium hypochlorite treatment; HPA, heat + hydrogen peroxide + acetic acid treatment. SA; *S*. Agona PARC 5, ST; *S*. Typhimurium LMFS-S-JF-001.
