# Supplementary figures and images for: Endogenous Metabolites Released by Sanitized Sprouting Alfalfa Seed Inhibit the Growth of Salmonella enterica
Source: mSystems. 2021 Feb 9;6(1):e00898-20. doi: 10.1128/mSystems.00898-20 (PMC7883538; doi:10.1128/mSystems.00898-20)

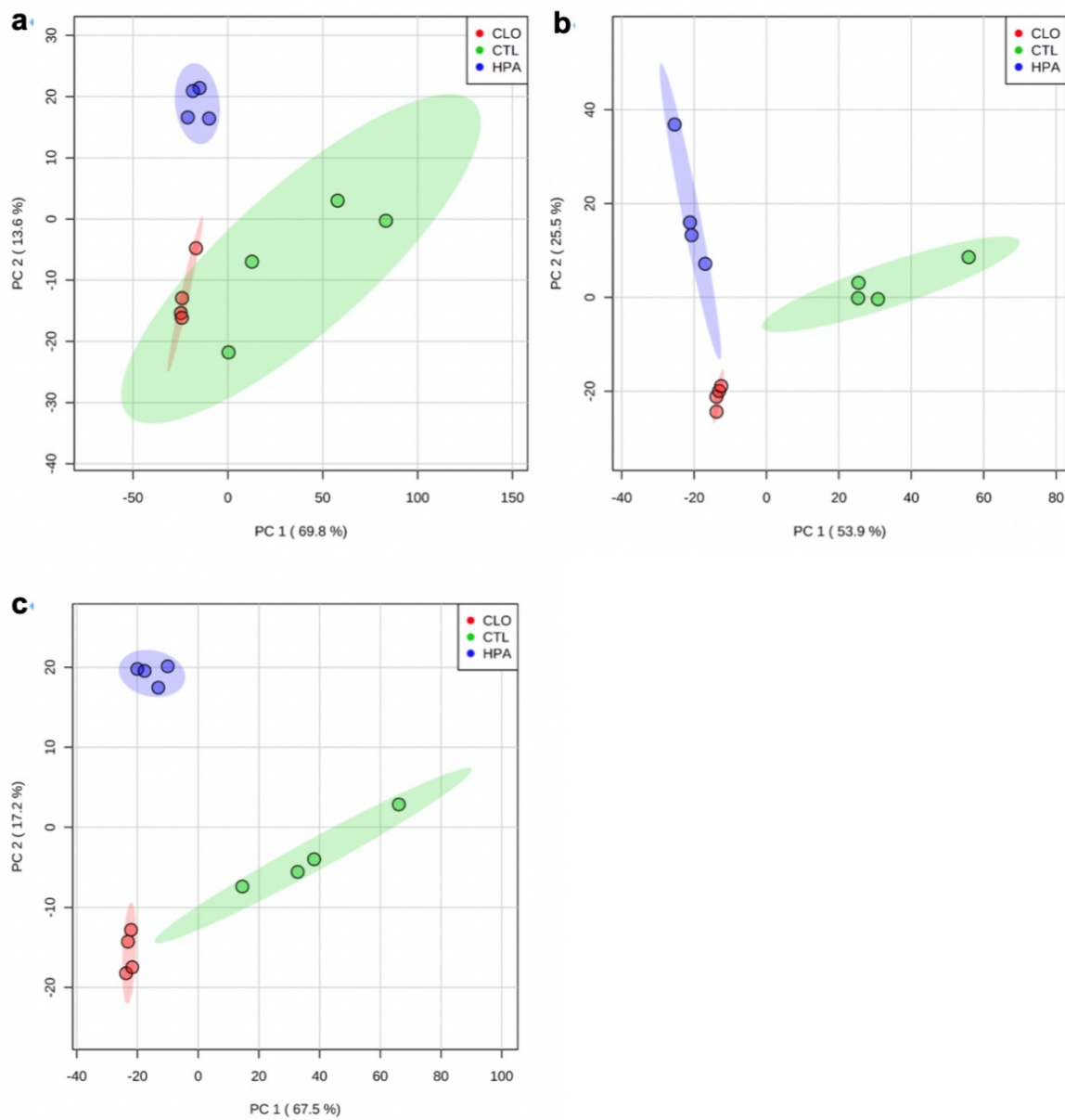

**Fig. S1.**

Supplement: FIG S1 [file mSystems.00898-20-sf001.pdf]
