# Supplementary material for: Endogenous Metabolites Released by Sanitized Sprouting Alfalfa Seed Inhibit the Growth of Salmonella enterica
Source: mSystems. 2021 Feb 9;6(1):e00898-20. doi: 10.1128/mSystems.00898-20 (PMC7883538; doi:10.1128/mSystems.00898-20)
Supplement: TABLE S2 [file mSystems.00898-20-st002.docx]

Table S2

| **Major pathway** | **Sub pathway** |  | **Increased metabolites** |  | **Decreased metabolites** |
| --- | --- | --- | --- | --- | --- |
| Amino acid | Serine family (phosphoglycerate derived) |  | - |  | serine |
|  | Aromatic amino acid metabolism (PEP derived) |  | - |  | shikimate |
|  |  |  |  |  | tryptophan |
|  |  |  |  |  | kynurenine |
|  |  |  |  |  | tyramine |
|  |  |  |  |  | 3-(4-hydroxyphenyl) propionate |
|  | Aspartate family (OAA derived) |  | N-acetylasparagine |  | aspartate |
|  |  |  |  |  | 2-aminoadipate |
|  |  |  |  |  | 6-oxopiperidine-2-carboxylate |
|  |  |  |  |  | methionine sulfoxide |
|  | Glutamate family (alpha-ketoglutarate derived) |  | - |  | 1-methyl-4-imidazoleacetate |
|  |  |  |  |  | carboxyethyl-GABA |
|  |  |  |  |  | glutamate |
|  |  |  |  |  | histidine betaine (hercynine) |
|  |  |  |  |  | stachydrine |
|  |  |  |  |  | homostachydrine |
|  | Amines and polyamines |  | - |  | N^1^, N^8^-acetylspermidine |
|  | Glutathione metabolism |  | - |  | ophthalmate |
|  |  |  |  |  | 5-oxoproline |
| Carbohydrate | Glycolysis |  | - |  | pyruvate |
|  | TCA cycle |  | - |  | citrate |
|  |  |  |  |  | malate |
|  | Photorespiration |  | oxalate (ethanedioate) |  | tartarate |
|  | Inositol metabolism |  | inositol 1-phosphate (I1P) |  | - |
|  | Sucrose, glucose, fructose metabolism |  | fucitol |  | galactinol |
|  |  |  | maltose |  | trehalose |
|  |  |  | verbascose |  | gluconate |
| Lipids | Free fatty acid |  | laurate (12:0) |  | - |
|  |  |  | docosadienoate (22:2n6) |  |  |
|  | Fatty acid, hydroxy |  | 3-hydroxymyristate |  | 2-hydroxyheptanoate |
|  |  |  | 3-hydroxybehenate |  | 3-hydroxyoctanoate |
|  |  |  |  |  | 8-hydroxyoctanoate |
|  |  |  |  |  | 2-hydroxydecanoate |
|  | Fatty acid, Dicarboxylate |  | - |  | adipate (C6-DC) |
|  |  |  |  |  | pimelate (C7-DC) |
|  |  |  |  |  | suberate (C8-DC) |
|  |  |  |  |  | azelate (C9-DC) |
|  |  |  |  |  | sebacate (C10-DC) |
|  |  |  |  |  | undecanedioate (C11-DC) |
|  |  |  |  |  | dodecanedioate (C12-DC) |
|  |  |  |  |  | dodecenedioate (C12:1-DC) |
|  | Fatty acid conjugate |  | - |  | acetylcarnitine (C2) |
|  | Choline metabolism |  | - |  | choline |
| Cofactors, Prosthetic Groups, Electron Carriers | CoA metabolism |  | - |  | pantothenate |
|  | Carnitine metabolism |  | - |  | deoxycarnitine |
|  | Ascorbate metabolism |  | - |  | ascorbate (Vitamin C) |
|  |  |  |  |  | threonate |
|  | Thiamine metabolism |  | - |  | 5-(2-Hydroxyethyl)-4-methylthiazole |
|  | Vitamin B metabolism (B6 or B12) |  | - |  | pyridoxate |
|  |  |  |  |  | pyridoxine (Vitamin B6) |
| Nucleotide | Purine metabolism |  | adenosine-2',3'-cyclic monophosphate |  | allantoic acid |
|  |  |  |  |  | allantoin |
|  |  |  |  |  | guanine |
|  |  |  |  |  | urate |
|  |  |  |  |  | xanthosine |
|  |  |  |  |  | 8-hydroxyguanine |
|  | Pyrimidine metabolism |  | - |  | 5,6-dihydrouridine |
|  |  |  |  |  | 5-methylcytidine |
|  |  |  |  |  | cytidine 2' or 3'-monophosphate |
|  |  |  |  |  | 2'-O-methylcytidine |
| Hormone metabolism | Abscisic acid metabolism |  | - |  | abscisate |
|  | Auxin metabolism |  | - |  | indoleacetylaspartate |
|  |  |  |  |  | indole-3-carboxylic acid |
| Secondary metabolism | Alkaloids |  | caffeine |  | salidroside |
|  | Benzenoids |  | - |  | 2,4,6-trihydroxybenzoate |
|  |  |  |  |  | 4-hydroxybenzoate |
|  |  |  |  |  | gentisic acid-5-glucoside |
|  |  |  |  |  | hydroquinone beta-D-glucopyranoside |
|  |  |  |  |  | salicylate |
|  | Fatty acid and sugar derivatives |  | - |  | galactarate (mucic acid) |
|  | Flavonoids |  | daidzein |  | dihydroquercetin |
|  |  |  | formononetin |  | quercetin 3-glucoside |
|  |  |  | liquiritigenin |  | kaempferol 3-O-glucoside/galactoside |
|  | Phenylpropanoids |  | - |  | 4-hydroxycinnamate |
|  |  |  |  |  | ferulate |
|  |  |  |  |  | sinapate |
|  |  |  |  |  | syringic acid |
|  |  |  |  |  | vanillate |
|  | Siderophores |  | - |  | deoxymugineic acid |
|  | Terpenoids |  | soyasaponin I |  | mevalonate |
|  |  |  |  |  | soyasaponin III |
| Xenobiotics | Chemicals |  | succinimide |  | trimethylamine N-oxide |
| Partially Characterized  Molecules | Partially Characterized Molecules |  | - |  | glucuronide of C_12_H_22_O_4_ (1) |
|  |  |  |  |  | glucuronide of C_12_H_22_O_4_ (2) |

Abbreviations: CTL, no treated control; CLO, sodium hypochlorite treatment; HPA, heat + hydrogen peroxide + acetic acid treatment.
