# Supplementary material for: Endogenous Metabolites Released by Sanitized Sprouting Alfalfa Seed Inhibit the Growth of Salmonella enterica
Source: mSystems. 2021 Feb 9;6(1):e00898-20. doi: 10.1128/mSystems.00898-20 (PMC7883538; doi:10.1128/mSystems.00898-20)
Supplement: TABLE S3 [file mSystems.00898-20-st003.docx]

Table S3

| **Metabolic Pathway** | **Metabolite Name** | **SA** | **ST** |
| --- | --- | --- | --- |
| Serine family (phosphoglycerate derived) | sulfate | 1.32 | 2.40 |
|  | cysteine sulfinic acid | 2.63 | 2.58 |
| Aromatic amino acid metabolism (PEP derived) | kynurenine | 1.38 | 1.64 |
|  | phenylpyruvate | 4.53 | 6.02 |
|  | 4-hydroxyphenylpyruvate | 8.83 | 8.22 |
|  | 3-(4-hydroxyphenyl) lactate | 1.51 | 1.66 |
| Aspartate family (OAA derived) | threonine | 1.24 | 1.97 |
|  | 2-aminoadipate | 1.63 | 2.26 |
|  | N6, N6, N6-trimethyllysine | 1.90 | 2.52 |
|  | N-acetylmethionine | 2.80 | 3.14 |
| Glutamate family (alpha-ketoglutarate derived) | 1-methyl-4-imidazoleacetate | 1.30 | 1.69 |
|  | argininosuccinate | 1.56 | 1.60 |
|  | dimethylarginine (SDMA + ADMA) | 1.86 | 2.34 |
|  | gamma-aminobutyrate (GABA) | 1.49 | 3.29 |
|  | histamine | 3.82 | 19.98 |
|  | trans-4-hydroxyproline | 1.25 | 1.86 |
|  | trans-urocanate | 1.72 | 2.46 |
|  | N-monomethylarginine | 1.78 | 2.17 |
|  | N-acetylhistamine | 1.47 | 2.08 |
| Branched Chain Amino Acids (pyruvate derived) | valine | 1.42 | 2.01 |
| Amines and polyamines | putrescine | 1.63 | 1.98 |
|  | feruloylputrescine | 1.45 | 1.70 |
|  | 1,3-diaminopropane | 1.67 | 1.72 |
| Glutathione metabolism | glutathione, oxidized (GSSG) | 6.50 | 7.14 |
|  | gamma-glutamylvaline | 1.94 | 3.49 |

Abbreviations: HPA, heat + hydrogen peroxide + acetic acid treatment; SA, *S*. Agona PARC 5; ST, *S*. Typhimurium LMFS-S-JF-001.
