# Supplementary material for: Endogenous Metabolites Released by Sanitized Sprouting Alfalfa Seed Inhibit the Growth of Salmonella enterica
Source: mSystems. 2021 Feb 9;6(1):e00898-20. doi: 10.1128/mSystems.00898-20 (PMC7883538; doi:10.1128/mSystems.00898-20)
Supplement: TABLE S4 [file mSystems.00898-20-st004.docx]

Table S4

| **Metabolite Name** | **SA** | **ST** |
| --- | --- | --- |
| 1-linoleoyl-2-linolenoyl-GPA (18:2/18:3) | 0.11 | 0.24 |
| 1-palmitoyl-2-linoleoyl-GPC (16:0/18:2) | 0.18 | 0.36 |
| 1-palmitoyl-2-oleoyl-GPC (16:0/18:1) | 0.18 | 0.25 |
| 1-stearoyl-2-linoleoyl-GPC (18:0/18:2) | 0.15 | 0.21 |
| 1-palmitoyl-2-alpha-linolenoyl-GPC (16:0/18:3n3) | 0.18 | 0.36 |
| 1-palmitoyl-2-oleoyl-GPE (16:0/18:1) | 0.04 | 0.07 |
| 1,2-dioleoyl-GPE (18:1/18:1) | 0.03 | 0.02 |
| 1-stearoyl-2-linoleoyl-GPE (18:0/18:2) | 0.02 | 0.01 |
| 1,2-dipalmitoyl-GPE (16:0/16:0) | 0.06 | 0.1 |
| 1,2-dilinoleoyl-GPE (18:2/18:2) | 0.05 | 0.06 |

Abbreviations: HPA, heat + hydrogen peroxide + acetic acid treatment; SA, *S*. Agona PARC 5; ST, *S*. Typhimurium LMFS-S-JF-001.
