# Supplementary material for: Endogenous Metabolites Released by Sanitized Sprouting Alfalfa Seed Inhibit the Growth of Salmonella enterica
Source: mSystems. 2021 Feb 9;6(1):e00898-20. doi: 10.1128/mSystems.00898-20 (PMC7883538; doi:10.1128/mSystems.00898-20)
Supplement: TABLE S5 [file mSystems.00898-20-st005.pdf]

Table S5

	Hours of germination					
	**0**	**4**	**8**	**12**	**24**	**144 (6 days)**
**Control**	4.67±0.76 ^{a}	4.91±0.99 ^{a}	5.17±0.30 ^{a}	7.87±0.06 ^{a}	9.05±0.54 ^{a}	7.53±0.14 ^{a}
**CLO treatment**	<1 ^{b}	<1 ^{b}	<1 ^{d}	3.49±0.31 ^{c}	7.69±0.16 ^{b}	7.23±0.30 ^{a}
**HPA treatment**	<1 ^{b}	<1 ^{b}	2.32±0.23 ^{c}	2.91±0.12 ^{d}	6.28±0.28 ^{c}	5.84±0.11 ^{b}
**CLO-CLO**	<1 ^{b}	<1 ^{b}	<1 ^{d}	4.18±0.33 ^{b}	5.13±0.16 ^{d}	6.10±0.14 ^{b}
**CLO-HPA**	<1 ^{b}	<1 ^{b}	2.10±0.18 ^{c}	3.49±0.20 ^{cd}	4.38±0.28 ^{d}	5.24±0.13 ^{c}
**HPA-HPA**	<1 ^{b}	<1 ^{b}	3.29±0.27 ^{b}	4.15±0.20 ^{b}	4.88±0.35 ^{d}	4.87±0.07 ^{c}
**HPA-CLO**	<1 ^{b}	<1 ^{b}	<1 ^{d}	3.94±0.08 ^{bc}	4.82±0.35 ^{d}	5.88±0.06 ^{b}

Data represent means ± standard deviations. Means with the same lowercase letter in the same column are not significantly different ($P \geq 0.05$).
CLO-CLO, Injection of metabolites from CLO treated alfalfa seeds on CLO treated alfalfa seeds inoculated with *S.* Agona PARC 5; CLO-HPA, Injection of metabolites from CLO treated alfalfa seeds on HPA treated alfalfa seeds inoculated with *S.* Agona PARC 5; HPA-HPA, Injection of metabolites from HPA treated alfalfa seeds on HPA treated alfalfa seeds inoculated with *S.* Agona PARC 5; HPA-CLO; Injection of metabolites from HPA treated alfalfa seeds on CLO treated alfalfa seeds inoculated with *S.* Agona PARC 5.
Abbreviations: CTL, no treated control; CLO, sodium hypochlorite treatment; HPA, heat + hydrogen peroxide + acetic acid treatment.
