# Supplementary material for: Endogenous Metabolites Released by Sanitized Sprouting Alfalfa Seed Inhibit the Growth of Salmonella enterica
Source: mSystems. 2021 Feb 9;6(1):e00898-20. doi: 10.1128/mSystems.00898-20 (PMC7883538; doi:10.1128/mSystems.00898-20)
Supplement: TABLE S6 [file mSystems.00898-20-st006.pdf]

Table S6

	Hours of germination					
	0	4	8	12	24	144 (6 days)
**Control**	3.44±0.71 ^{a}	4.54±0.94 ^{a}	5.02±0.48 ^{a}	5.91±0.14 ^{a}	7.28±0.29 ^{a}	7.44±0.45 ^{a}
**CLO treatment**	<1 ^{b}	<1 ^{b}	3.08±0.11 ^{b}	3.73±0.12 ^{bc}	5.47±0.36 ^{b}	6.19±0.28 ^{a}
**HPA treatment**	<1 ^{b}	<1 ^{b}	2.57±0.39 ^{bc}	3.17±0.23 ^{c}	4.75±0.19 ^{b}	3.91±0.32 ^{c}
**CLO-CLO**	<1 ^{b}	<1 ^{b}	3.15±0.19 ^{b}	3.94±0.55 ^{b}	5.17±0.42 ^{b}	5.87±0.23 ^{a}
**CLO-HPA**	<1 ^{b}	<1 ^{b}	1.84±0.21 ^{c}	3.73±0.34 ^{bc}	5.18±0.37 ^{b}	4.27±0.29 ^{c}
**HPA-HPA**	<1 ^{b}	<1 ^{b}	2.72±0.41 ^{b}	3.94±0.08 ^{b}	4.82±0.35 ^{b}	3.44±0.31 ^{d}
**HPA-CLO**	<1 ^{b}	<1 ^{b}	3.05±0.14 ^{b}	3.99±0.09 ^{b}	5.07±0.18 ^{b}	5.08±0.16 ^{b}

Data represent means ± standard deviations. Means with the same lowercase letter in the same column are not significantly different ($P \geq 0.05$).
CLO-CLO, Inoculation of metabolites from CLO treated alfalfa seeds on CLO treated alfalfa seeds inoculated with *S.* Typhimurium LMFS-S-JF-001; CLO-HPA, Inoculation of metabolites from CLO treated alfalfa seeds on HPA treated alfalfa seeds inoculated with *S.* Typhimurium LMFS-S-JF-001; HPA-HPA, Inoculation of metabolites from HPA treated alfalfa seeds on HPA treated alfalfa seeds inoculated with *S.* Typhimurium LMFS-S-JF-001; HPA-CLO, Inoculation of metabolites from HPA treated alfalfa seeds on CLO treated alfalfa seeds inoculated with *S.* Typhimurium LMFS-S-JF-001.
Abbreviations: CTL, no treated control; CLO, sodium hypochlorite treatment; HPA, heat + hydrogen peroxide + acetic acid treatment.
