# Supplementary material for: Endogenous Metabolites Released by Sanitized Sprouting Alfalfa Seed Inhibit the Growth of Salmonella enterica
Source: mSystems. 2021 Feb 9;6(1):e00898-20. doi: 10.1128/mSystems.00898-20 (PMC7883538; doi:10.1128/mSystems.00898-20)
Supplement: TABLE S7 [file mSystems.00898-20-st007.pdf]

Table S7

Supplement	Con.	Hours of germination						
		0	4	8	12	24	48	144 (6 days)
**Control**	0 ppm	4.67±0.76 ^{a}	4.91±0.99 ^{ab}	5.17±0.30 ^{bcd}	7.87±0.06 ^{a}	9.05±0.54 ^{a}	7.28±0.13 ^{b}	7.53±0.14 ^{a}
**Spermidine**	10 ppm	4.14±0.27 ^{a}	4.20±0.15 ^{b}	4.41±0.43 ^{e}	7.39±0.05 ^{bc}	8.06±0.53 ^{b}	7.06±0.08 ^{a}	6.93±0.12 ^{bc}
	100 ppm	4.11±0.21 ^{a}	4.24±0.19 ^{b}	4.63±0.55 ^{de}	7.61±0.25 ^{abc}	8.17±0.43 ^{b}	7.09±0.15 ^{a}	6.81±0.13 ^{bc}
	500 ppm	4.08±0.13 ^{a}	4.30±0.14 ^{b}	4.79±0.36 ^{de}	7.76±0.21 ^{ab}	8.91±0.09 ^{a}	7.11±0.13 ^{a}	6.76±0.26 ^{c}
**Agmatine**	10 ppm	4.12±0.42 ^{a}	5.11±0.10 ^{ab}	5.70±0.21 ^{abc}	6.61±0.27 ^{e}	8.55±0.25 ^{a}	8.11±0.18 ^{a}	7.07±0.15 ^{b}
	100 ppm	4.15±0.35 ^{a}	5.32±0.06 ^{ab}	6.00±0.29 ^{ab}	7.12±0.21 ^{bcd}	8.57±0.29 ^{a}	8.09±0.16 ^{a}	6.99±0.10 ^{bc}
	500 ppm	4.17±0.48 ^{a}	5.64±0.28 ^{a}	6.49±0.19 ^{a}	7.69±0.22 ^{cde}	8.60±0.39 ^{a}	8.05±0.09 ^{a}	6.96±0.13 ^{bc}
**N^1- and N^8-acetylspermidine**	10 ppm	4.33±0.25 ^{a}	4.46±0.19 ^{b}	5.08±0.18 ^{cd}	6.66±0.39 ^{e}	8.07±0.25 ^{b}	6.65±0.31 ^{b}	6.01±0.25 ^{d}
	100 ppm	4.63±0.05 ^{a}	4.55±0.41 ^{ab}	5.29±0.08 ^{bcd}	6.88±0.14 ^{de}	8.00±0.30 ^{b}	6.15±0.27 ^{bc}	5.83±0.28 ^{e}
	500 ppm	4.60±0.08 ^{a}	4.45±0.39 ^{b}	5.51±0.16 ^{bcd}	7.05±0.23 ^{cde}	8.09±0.37 ^{b}	6.09±0.28 ^{c}	5.65±0.35 ^{e}

Data represent means ± standard deviations. Means with the same lowercase letter in the same column are not significantly different ($P \geq 0.05$).
