# Supplementary material for: Endogenous Metabolites Released by Sanitized Sprouting Alfalfa Seed Inhibit the Growth of Salmonella enterica
Source: mSystems. 2021 Feb 9;6(1):e00898-20. doi: 10.1128/mSystems.00898-20 (PMC7883538; doi:10.1128/mSystems.00898-20)
Supplement: TABLE S8 [file mSystems.00898-20-st008.pdf]

Table S8

Supplement	Con.	Hours of germination						
		0	4	8	12	24	48	144 (6 days)
**Control**	0 ppm	3.15±0.50 [a]	4.54±0.94 [a]	5.02±0.76 [ab]	5.92±0.42 [ab]	7.28±0.03 [a]	7.17±0.17 [abc]	6.11±0.25 [a]
**Spermidine**	10 ppm	3.38±0.44 [a]	3.86±0.38 [a]	4.41±0.19 [bcd]	5.39±0.27 [b]	7.47±0.16 [ab]	6.95±0.16 [cd]	5.67±0.22 [bcd]
	100 ppm	3.23±0.40 [a]	3.95±0.08 [a]	4.27±0.26 [cde]	5.26±0.32 [b]	7.70±0.34 [ab]	7.14±0.18 [abc]	5.48±0.13 [cde]
	500 ppm	3.10±0.04 [a]	4.17±0.13 [a]	4.60±0.27 [abcd]	5.80±0.14 [ab]	7.95±0.08 [b]	7.12±0.10 [abc]	5.22±0.20 [de]
**Agmatine**	10 ppm	3.45±0.46 [a]	3.55±0.21 [a]	3.76±0.12 [e]	5.40±0.31 [b]	7.53±0.40 [ab]	7.46±0.20 [a]	6.07±0.20 [b]
	100 ppm	3.61±0.40 [a]	3.56±0.29 [a]	3.95±0.05 [de]	5.66±0.19 [ab]	7.63±0.38 [ab]	7.32±0.39 [ab]	5.93±0.33 [b]
	500 ppm	3.54±0.29 [a]	3.55±0.35 [a]	4.29±0.19 [cde]	6.06±0.20 [a]	7.76±0.26 [b]	7.27±0.19 [abc]	6.05±0.26 [b]
**N^1- and N^8-acetylspermidine**	10 ppm	4.29±0.31 [a]	3.87±0.59 [a]	4.63±0.17 [abc]	5.45±0.25 [ab]	7.13±0.24 [a]	6.99±0.15 [bcd]	5.61±0.20 [bc]
	100 ppm	4.13±0.24 [a]	4.31±0.34 [a]	4.90±0.16 [abc]	5.46±0.12 [ab]	7.23±0.11 [a]	6.95±0.12 [cd]	5.23±0.17 [bc]
	500 ppm	4.26±0.21 [a]	4.21±0.29 [a]	5.21±0.16 [a]	5.66±0.19 [ab]	7.27±0.07 [a]	6.76±0.24 [d]	5.09±0.14 [e]

Data represent means ± standard deviations. Means with the same lowercase letter in the same column are not significantly different ($P \geq 0.05$).
